# Boosting fluorescence efficiency via filling technique prepared photonic crystal composites

**DOI:** 10.1038/s41598-025-04296-7

**Published:** 2025-06-06

**Authors:** Yutao Qin, Xiang Zhao, Yiran Wang, Jiaxiang He, Zheng Zhu, Tianzhuo Zhao, Guoyan Dong

**Affiliations:** 1 Center of Materials Science and Optoelectronics Engineering, School of Opto-Electronics, University of Chinese Academy of Sciences, Beijing, 100049 China; 2 School of Physics, Beijing Institute of Technology, Beijing, 100081 China

**Keywords:** Porous photonic crystal, Fluorescence enhancement, Localized surface plasmon resonance, Photonic band gap, High density of state, Photonic crystals, Photonic crystals

## Abstract

Au-doped photonic crystals offer considerable potential for boosting optical signals, however, precisely controlling the distance between luminescent particles and Au nanoparticles (NPs) faces severe challenges. We proposed a “filling” technique to prepare porous Au-doped inverse-opal PC (IOPC) with encapsulated Au NPs uniformly dispersing in insulating silica. The effective separation between Au NPs and infiltrated luminescent quantum dots successfully addresses the issue of fluorescence quenching, enhancing the photoluminescence intensity by 106-fold. Additionally, the double-layer IOPC-OPC composite, integrating an Au-doped IOPC and an opal photonic crystal (OPC) completely reflecting excitation or emission light, significantly improves the fluorescence intensity to 242-fold, far superior to the published counterparts. This synergy of localized surface plasmon resonance, high density of state, and photonic band gap in the IOPC-OPC composite offers an effective and low-loss approach for the precise modulation and amplification of photoluminescence. This strategy is crucial for the development of next-generation optical devices with improved sensitivity and stability.

## Introduction

Photonic crystals (PCs) with periodic dielectric structures can manipulate light propagation or prohibit light within specific frequency ranges. Three-dimensional opal-type PCs, inspired by natural photonic structures found in biological systems, have been extensively studied due to their facile self-assembly fabrication process.^1^Porous inverse-opal photonic crystals (IOPCs) can be achieved by infilling different functional materials in the void of opal PC (OPC) templates^2,3^, and then removing the templates by calcination. The porous IOPCs of rich structural colors have been applied to improve photocatalytic performance^4,5^, develop various sensors of pH^6,7^, stress^8,9^, current^10,11^, or detect multiple biomolecular substances^12,13^. High density of state^14,15^(DOS) near the photonic band edges of PCs plays a significant role in improving light-matter interaction. In recent years, diverse PCs have been employed to tune the fluorescence properties of various luminescent materials. Titanium-oxide IOPCs^16^ were successfully fabricated to enhance the fluorescence intensity of incorporating up-conversion particles by 43-fold. Double-layer OPCs^17^ were observed to enhance the fluorescence of Nile Red dye. Three-layer OPC structure^18^ of double heterojunctions with carbon quantum dots (QDs) infilled the middle layer resulted in great fluorescence enhancement. However, it is difficult for luminescent materials to penetrate close-packed OPCs to enhance light-matter interaction, moreover, the heterojunction structures complicate the sample preparation process.

Localized surface plasmon resonance (LSPR) of metal NPs facilitates improving luminescence efficiency^19,20^, but physical contact and close distance between metal and fluorescent materials lead to fluorescence quenching^21^, impeding fluorescence enhancement. Silica coating^22^ had been utilized to mitigate the quenching phenomenon through an intricate preparation process challenging to execute. Other available core-shell structures^23,24^ were also prepared to address various problems in the field of detection and sensing. Combining the flexible photon band gap (PBG) characteristics^25,26^ of PCs with the LSPR effect of metal NPs presents a powerful advantage for enhancing fluorescence and regulating luminescence behaviors. Fluorescent substances capped with Au nanoclusters were infilled into PCs to observe the fluorescent signal^27^. Erola et al. infiltrated IOPCs into metal NP colloids directly^28^. Nano-silver was deposited onto the surface of perovskite OPCs to increase luminescence intensity^29^. Magnetron sputtering technique was employed to deposit silver NPs onto the PC surface, achieving multiple orders of intensity enhancement^30^. Furthermore, the “one-pot” method was widely used to prepare Au-doped porous silica IOPCs^31–33^ to enhance hue and saturation, but surface-aggregation of Au NPs hindered their role in fluorescence enhancement.

In this work, we first proposed a “filling” technique to synthesize porous silica IOPCs with encapsulated Au NPs uniformly distributing. The distance between Au NPs and luminescent QDs can be precisely tuned to address the issue of fluorescence quenching. Significant fluorescence enhancement by 242-fold has been realized via an IOPC-OPC composite configuration. This strategy can also be extended to other luminescent materials, to improve photocatalytic effect, Biosensing sensitivity, imaging resolution, display clarity, and so on.

## Results and discussion

**Fig. 1:**
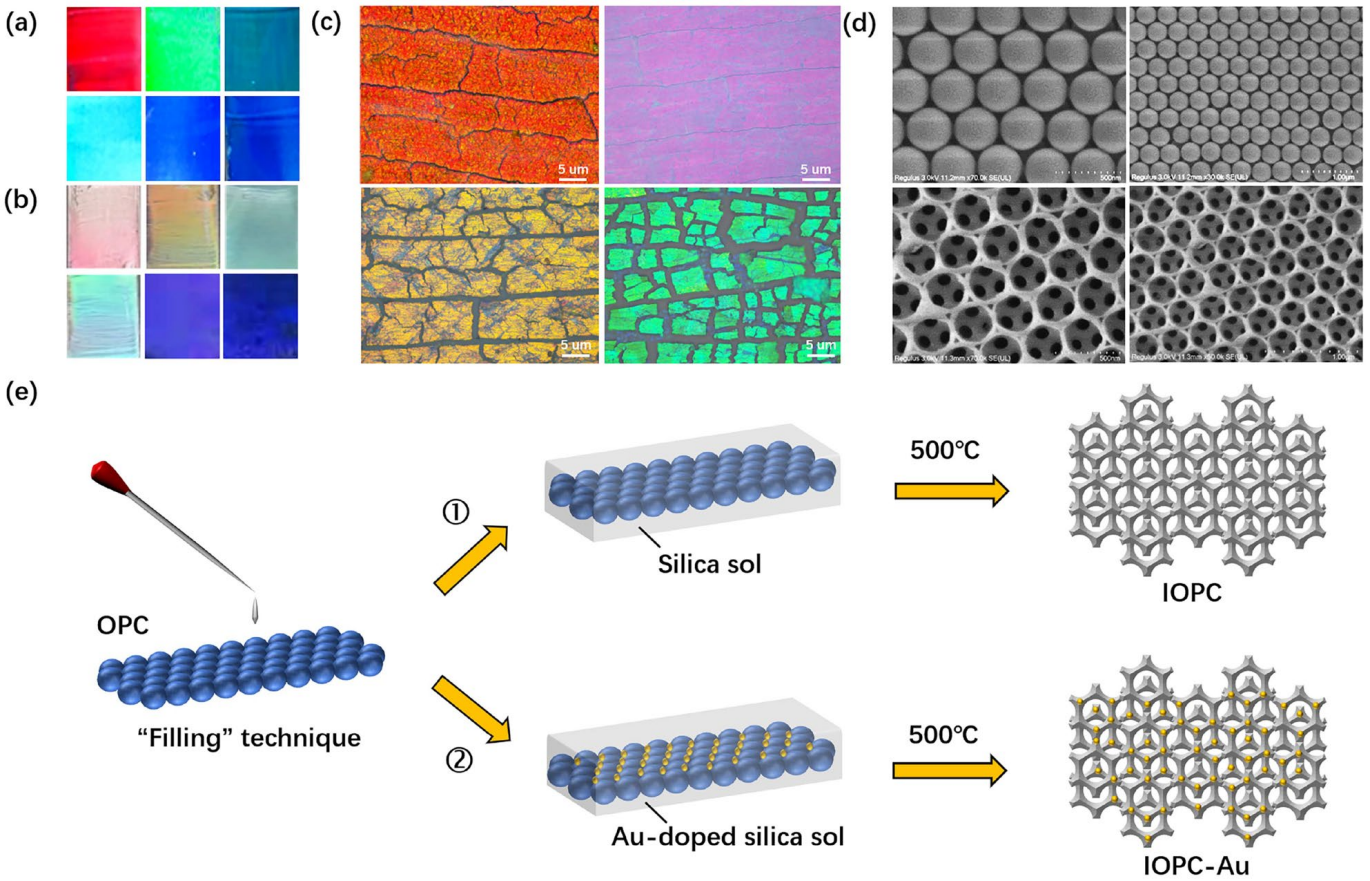
Structural color modulation and morphology. (**a**) OPCs composed of PS microspheres with diameters of 285, 240, 220, 210, 200, and 165 nm (by row, left to right), showing tunable PBG-derived colors. (**b**) Silica IOPCs with lattice constants of 430, 360, 340, 280, 250, and 240 nm (by row, left to right), exhibiting PBG-dependent color shifts. (**c**) Optical micrographs and (**d**) SEM images of OPC (top) and IOPC (bottom) samples. (**e**) Schematic of the silica IOPC fabrication process: (1) PS template infiltration with silica precursors, (2) Au-doped silica IOPC for plasmonic enhancement.

Self-assembly technique was employed to fabricate OPCs and IOPCs using the polystyrene (PS) microspheres of different sizes, exhibiting diverse structural colors, as illustrated in Fig. 1a and 1b. The microscopic and scanning electron microscope (SEM) images of prepared OPCs and IOPCs are depicted in Fig. 1c and 1d, respectively, providing a glimpse into the microstructure of the PC samples in different magnifications. Figure 1e outlines two schemes for synthesizing IOPCs and Au-doped IOPCs through the procedures of sol-gel synthesis and high-temperature calcination, where the OPC sample serves as a sacrificial template. The porous architectures of IOPCs facilitate the infiltration and encapsulation of fluorescent NPs. The correlation between the diameter of PS microspheres and the PBG wavelength of the self-assembled OPC can be expressed as follows^14^:

1 $$\begin{aligned} \lambda =2\sqrt{2/3}d\cdot \sqrt{\sum {n_{i}^{2}}\phi _{i}-{\sin ^{2}\theta }} \end{aligned}$$

where *d* is the diameter of constituent PS microspheres, *n* and $\phi$ represent the refractive index and filling rate of the OPC, and $\theta$ represents incident angle. Here, the refractive index of the PS microspheres is *n*=1.58, with the filling rate $\phi$=0.74, and $\theta$=0. The experimentally measured linear relationship between the diameter of the constituent PS microspheres and the PBG centeral wavelength of prepared OPC exhibits agreement with that of theoretical calculations (Supplementary Fig. S1), as predicted by the modified Bragg’s law for first-order diffraction (Eq. 1). This validates the precision of our sample preparation in tuning the optical properties of PCs.

**Fig. 2:**
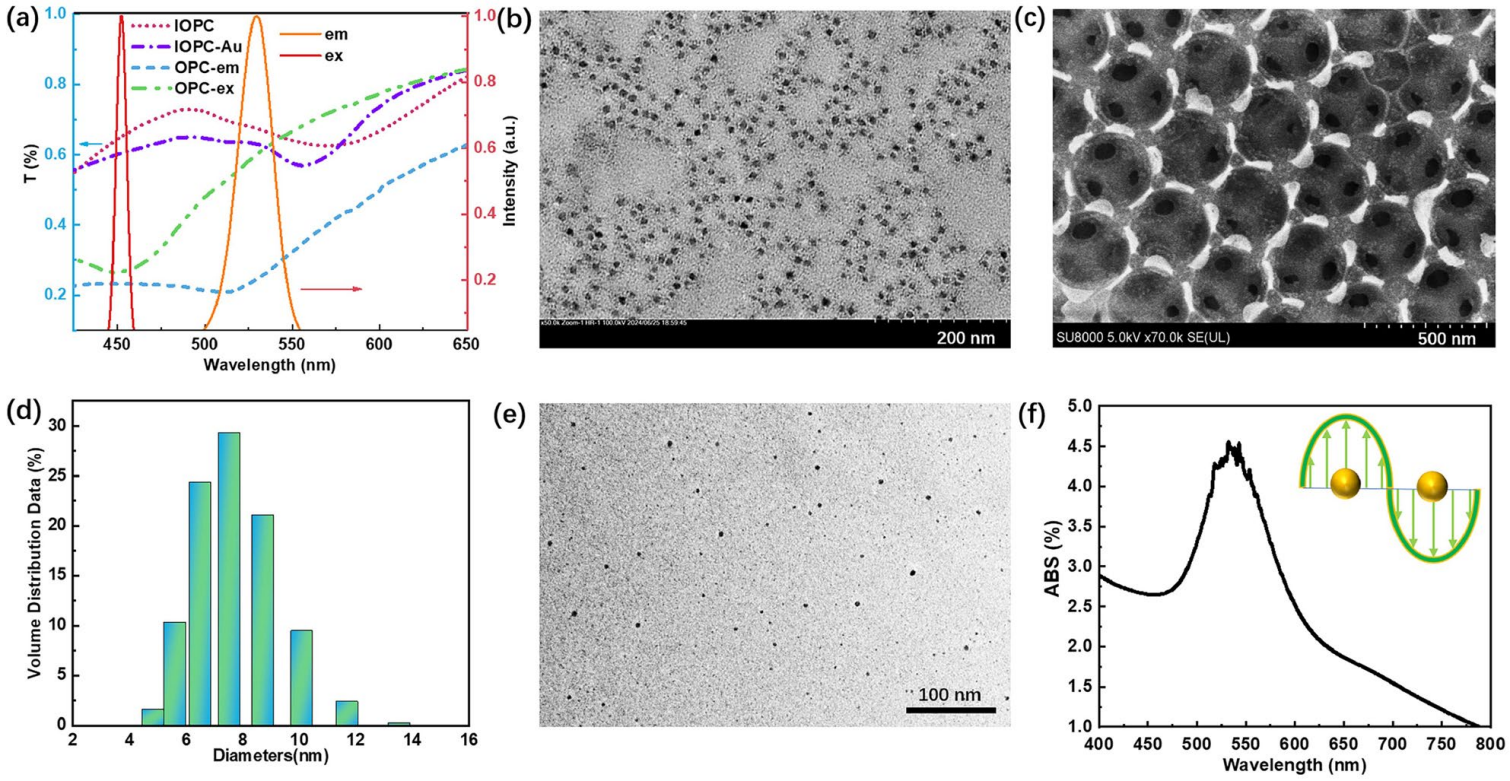
(**a**) Transmission spectra of prepared OPCs and IOPCs, and normalized excitation (red solid line, ex) and emission (orange solid line, em) spectra of CdSe QDs. (**b**) TEM image of monodisperse CdSe QDs with diameters of 8 ± 2 nm. (**c**) SEM image of QDs-filled IOPC. (**d**) Size distribution histogram and (**e**) TEM image of synthesized Au NPs. (**f**) Absorption spectrum of Au NP suspension, showing a LSPR peak at 527 nm for spectral overlap with QD emission.

The photoluminescence (PL) material CdSe QDs were employed as the fluorescent material due to their high brightness and tunable emission intensity. The red and yellow solid lines in Fig. 2a demonstrate that the spectral characteristics of CdSe QDs exhibit excitation (ex) and emission (em) peaks centered at 450 nm and 527 nm, respectively. We tailored the excitation and emission PBG wavelengths of the OPCs by selecting the PS microspheres of 200 nm and 260 nm diameters. The PBGs of prepared OPC-ex and OPC-em samples are localized near the excitation peak (450 nm) and the emission peak (527 nm) of CdSe QDs, respectively. In the “filling” technique, we employed the PS microparticles of the diameter 340 nm to prepare the IOPC with its left band edge (pink dot line) covering the fluorescence wavelength of 527 nm. Further investigation found that the doped Au NPs led to a small blue shift of the IOPC’s PBG (purple dot-dash line), strengthening the high DOS near the band edge of the Au-doped IOPCs. The transmission electron microscope (TEM) image of the CdSe QDs (Fig. 2b) and the SEM image of the IOPC infiltrated by CdSe QDs (Fig. 2c) clarify that the QDs are small enough to infiltrate the porous IOPCs, attaching to the SiO_2_ wall. The band edges of the IOPC provided the high-DOS condition to improve light-matter interaction for fluorescence enhancement, and the drastic near-field LSPRs near the Au NPs significantly enhanced the luminescent efficiency of QDs. The detailed preparation procedures for OPCs and IOPCs have been explained in the Methods Section.

In the “filling” approach, the doped Au NPs were prepared in advance with a specific chemical reduction method: A 50 mL solution of 0.01 wt% chloroauric acid (HAuCl$\phantom{0}_4$) was prepared and heated to boiling. Subsequently, 1 mL of 30 wt% trisodium citrate solution was swiftly added to the boiling HAuCl$\phantom{0}_4$ solution and continuously stirred for 15-20 min. In this way, the size distribution of resulting Au NPs can be flexibly regulated by changing the mix ratio of two solutions. Ultrasonic dispersion continued for 30 min, to ensure the uniform distribution of Au NPs. The histogram in Fig. 2d reveals the measured size distribution of Au NPs, with an average diameter of 8±2 nm. The TEM image in Fig. 2e shows the morphologies of these synthesized Au NPs, leading to the absorption peak of LSPRs covering the fluorescence wavelength of CdSe QDs near 527 nm (Fig. 2f).

**Fig. 3:**
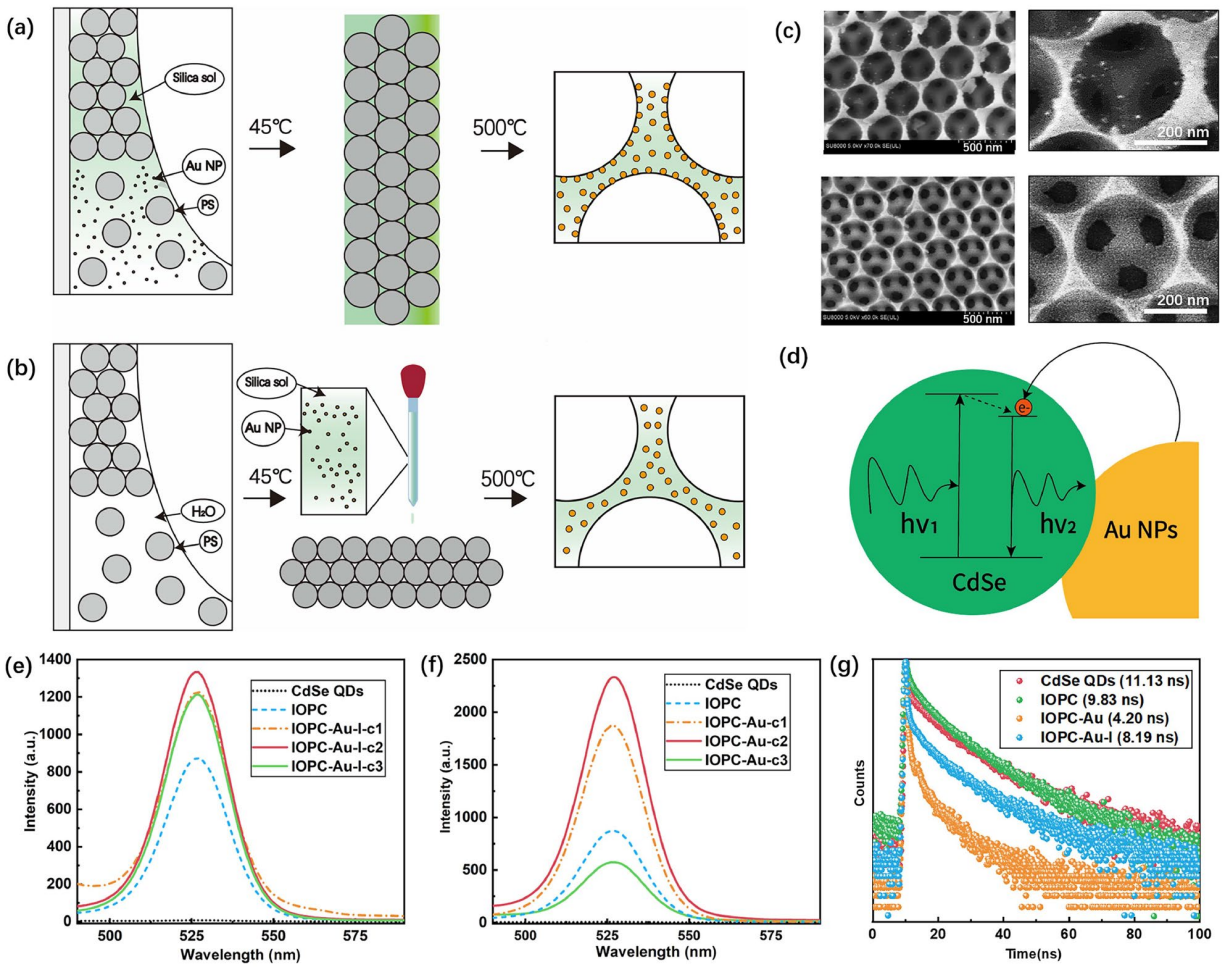
Fabrication strategies and plasmon-enhanced fluorescence of Au-doped IOPCs. (**a**) Schematics of “one-pot” synthesis and (**b**) “filling” method for porous Au-doped silica IOPCs. (**c**) SEM images and enlarged details of Au-doped IOPCs prepared by “one-pot” (top) and “filling” (bottom) techniques. (**d**)  Mechanism of LSPR effect enhancing QDs emission through near-field coupling. (**e**) Fluorescence spectra of CdSe QDs infiltrated in (**e**) “one-pot” and (**f**) “filling” IOPCs with varing volume ratios of Au-NPs to silica sol. (**g**) Time-resolved PL decays of CdSe QDs in free space (red), IOPC (green), “filling” Au-doped IOPC (orange), and “one-pot” Au-doped IOPC (blue).

The near-field enhancement of LSPR via uniform Au NPs plays an important role in modulating fluorescence emission. However, the close contact between metallic NPs and fluorescent materials leads to fluorescence quenching^34^, impeding the enhancement of fluorescent. The “filling” technique is proposed to prepare porous Au-doped IOPC samples and simultaneously address the issue of fluorescence quenching between Au NPs and infiltrated QDs, avoiding the intricate and laborious preparation process of Au@SiO$\phantom{0}_2$ core-shell fluorescent NPs^35^. As a comparison, we synthesized Au-doped IOPC using the common “one-pot” technique (IOPC-Au-I). Figure 3a depicts its self-assembly procedure. The pre-prepared hybrid sol of silica with Au NPs was added to the PS microsphere (*d*=360 nm) suspension together to form a mixed suspension. Pre-cleaned quartz substrates were inclined and dipped in the hybrid suspension. During the self-assembly process of over 12 hours, the Au NPs preferentially deposited on the surface of the PS microspheres due to the $\pi$-metal interaction^36^ between them. After high-temperature ($500^\circ \textrm{C}$) calcination, the PS microspheres were removed to obtain the porous silica IOPC. However, due to the collective Au NPs on the pore-wall surfaces, some residual fragments dispersed around, as the SEM image and enlarged detail shown in the top row of Fig. 3c. After infilling CdSe QDs into the porous IOPC, the energy transfer between the QDs-Au NPs system and the hot electrons of Au NPs injecting into the conduction bands of QDs (Fig. 3d) result in fluorescence enhancement or quenching^37^, depending on the QD-Au NP distance counterbalance. Figure 3e illustrates the PL spectra of the QDs-infiltrated “one-pot” IOPCs incorporated with Au NPs of different ratios (IOPC-Au-I), where IOPC-Au-I-c1, c2, and c3 represent the volume fractions of 0.2, 0.6, and 1 for Au sol relative to silica sol, respectively. Although the IOPCs incorporating with Au NPs via the “one pot” method exhibited enhanced fluorescence intensity, the aggregation of Au NPs on the porous surfaces induced significant fluorescence quenching through non-radiative energy transfer, substantially limiting the overall fluorescence enhancement efficiency.

Maximizing the DOS within the emission band is crucial for enhancing fluorescence intensity through the Purcell effect, hence, the PS microspheres of the diameter 340 nm were strategically selected to prepare the OPC templates in the “filling” approach, ensuring that the band edge of the resulting IOPC precisely overlaps with the emission peak of the QDs at 527 nm (Supplementary Fig. S4). The previously synthesized uniform Au NPs solution was mixed with the silica sol in a certain volume ratio, after ultrasonic dispersion for 30 min, to form uniformly mixed solution. The solution was gradually infiltrated into the preformed OPC template, followed by rapid gelation within approximately 1 min through acid-catalyzed condensation of the silica. This process effectively immobilized the Au NPs, preventing further migration and aggregation. This “filling” approach effectively avoided the undesirable $\pi$-metal attraction and subsequent deposition of Au NPs onto the PS microspheres’ surfaces in the “one-pot” approach, ensuring that the Au NPs were evenly distributed and completely wrapped within the silica matrix. The insulating silica isolated the Au NPs from the QDs, thereby preventing direct contact-induced fluorescence quenching through non-radiative energy transfer. Following the same calcination, we achieved the premium porous Au-doped IOPC, with the encapsulated Au NPs evenly dispersing in silica (Fig. 3b). In Fig. 3c, the bottom SEM images with the enlarged detail exhibits the morphology of smooth SiO$\phantom{0}_2$ wall encapsulating Au NPs.

In the “one-pot” approach, since the hydrochloric acid was added for the hydrolysis of ethyl silicate, the suspension composed of PS microspheres, Au NPs and silica-sol displayed acidic (pH=5.85). In the weakly acidic system, the electrostatic repulsion of negative charges was reduced or even eliminated, leading to the failure of electrostatic stabilization. Meanwhile, the robust $\pi$-metal interaction between the $\pi$-bonds of the benzene rings of PS microspheres and the free electrons of Au NPs led to their mutual attraction, and some Au NPs deposited onto the surface of the PS microspheres. This interaction is independent of the solution polarity. Figure 3d illustrates the schematic mechanism of LSPR-enhanced near-field emission of QDs under the effect of Au NPs, due to the Förster resonance energy transfer in a local field and hot electrons injected into the conduction bands of QDs with nonradiative energy transfer. The fluorescence efficiency of QDs can be precisely tuned by changing the doping concentration of Au NPs. Therefore, the concentration parameter of the Au NPs was optimized to achieve the maximal PL enhancement for the same quantitative QDs. Figure 3f illustrates the measured photoluminescence (PL) spectra of the same CdSe QDs filled into the “filling” IOPCs incorporated with different concentrations of Au NPs, where the IOPC-Au-c1, c2, and c3 denote the volume ratios 0.17, 0.33, and 1 of Au NPs solution to silica sol, respectively. The optimal fluorescence intensity of the “filling” IOPC achieved at the optimal concentration of c2=0.33 (Fig. 3f) is much larger than that of the “one-pot” IOPC achieved at the optimal case of c2=0.6 (Fig. 3e). As the doping ratio of Au NPs gradually increases, more embedded Au NPs approach the pore-wall of IOPCs. The strong local electric field induced by LSPR resonantly transfers energy to adjacent QDs, leading to the fluorescence enhancement of CdSe QDs, until the silica spacer layer is broken, fluorescence quenching occurs in the “filling” Au-doped IOPC, leading to the rapid fluorescence decay.

Under identical excitation conditions, the higher the quantum yield leads to stronger emitted fluorescence intensity, which is related to their fluorescence lifetime. Figure 3g depicts the measured fluorescence lifetimes of the same CdSe QDs filled in various IOPC configurations. The measured baseline fluorescence lifetime of CdSe QDs in free space is 11.13 ns. The short fluorescence lifetime of 9.83 ns of the undoped IOPC demonstrates that the effect of high DOS near PBG edge on the fluorescence enhancement is based on the increased rate of radiation transition. The shorter fluorescence lifetime of 8.19 ns via the “one-pot” Au-doped IOPC (IOPC-Au-I) confirms the positive influence of uniform Au NPs’ LSPR on fluorescence efficiency. In the “filling” Au-doped IOPC (IOPC-Au), the proper separation between Au NPs and CdSe QDs maximizes the LSPR impact on fluorescence enhancement, while preventing fluorescence quenching. The shortest fluorescence lifetime 4.2 ns agrees well with the case of optimal fluorescence intensity in Fig. 3f. It further verifies the “filling” technique provided a more practical and effective way to harness the synergistic luminescence enhancement effect arising from the high DOS of PCs and the LSPR of metallic NPs.

**Fig. 4:**
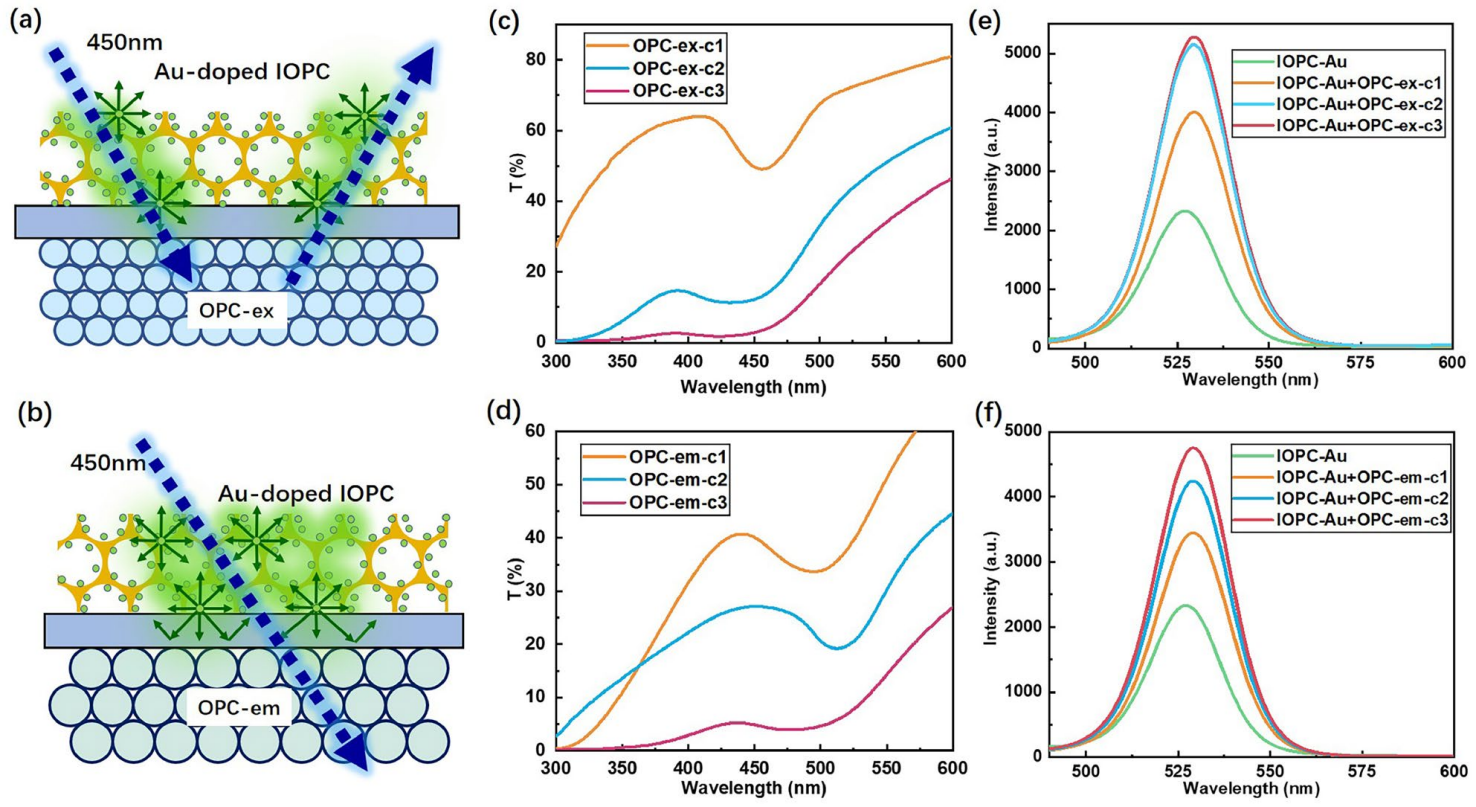
Schematics of IOPC-OPC double-layer composites integrating an Au-doped IOPC layer with (**a**) an OPC-ex reflective layer (450 nm PBG) or (**b**) an OPC-em reflective layer (527 nm PBG). Transmittance spectra of (**c**) OPC-ex and (**d**) OPC-em reflection layers prepared using PS microsphere suspensions (c1, c2, and c3) with concentrations of 1.25, 1.875, and 3.125 wt%. Fluorescence spectra of the double-layer IOPC-OPC composites incorporating (**e**) OPC-ex or (**f**) OPC-em reflectors with varing thickness.

The PL efficiency of CdSe QDs was remarkably enhanced through the synergetic effect of high-DOS and LSPR in the “filling” Au-doped IOPC structure. The appropriate thickness of the Au-doped IOPC facilitated QDs’ uniform infiltration and fluorescence enhancement, however, the isotropic fluorescence radiation and backside leakage losses reduced the forward PL efficiency of CdSe QDs. Therefore, we integrated the optimal “filling” Au-doped IOPC with an OPC to form a double-layer IOPC-OPC composite, leveraging the high reflectivity of the underlying OPC’s PBG near the excitation (450 nm) or emission (527 nm) wavelengths to prevent backside leakage. The PS microspheres of 200 nm and 260 nm diameters were employed to prepare the OPC-ex with a PBG at 450 nm and the OPC-em with a PBG at 527 nm. As the schematic shown in Fig. 4a, when the underlayer OPC-ex selectively reflects at the excitation wavelength (e.g., 450 nm), the incident photons undergo multiple scattering and reflection, leading to their spatial confinement within the QD-filled layer. This photonic concentration effect significantly enhances the excitation efficiency of QDs. As the schematic shown in Fig. 4b, when the underlayer OPC-em reflects all backward-leaking fluorescence at 527 nm, the enhanced local density of optical state (LDOS) in the upper IOPC-Au layer promotes spontaneous radiation and suppresses nonradiative recombination of the embedded QDs. The detected forward fluorescence intensity has more than doubled. Both of the aforementioned IOPC-OPC composite strategies significantly enhanced the forward PL intensity of CdSe QDs.

The thickness of the OPC layer can be tuned by changing the concentration of PS microsphere suspension in the self-assembly process. We prepared three types of PS microsphere suspensions (c1, c2, and c3) with concentrations of 1.25, 1.875, and 3.125 wt% to assemble the OPC-ex and OPC-em reflectors with diverse thicknesses. As the measured transmission spectra exhibit in Fig. 4c and 4d, the increasing concentration of the PS microsphere suspension led to the thickening OPC reflective layers with decreasing transmittance. We respectively integrated the OPC-ex and OPC-em reflectors with the optimal “filling” Au-doped IOPC layer (IOPC-Au) to form two types of IOPC-OPC composites (i.e. IOPC-Au+OPC-ex and IOPC-Au+OPC-em). Figures 4e and 4f exhibit the measured forward fluorescence spectra via the IOPC-OPC composites by combining the fixed optimal Au-doped IOPC-Au with the OPC-ex and the OPC-em layers of different thicknesses, respectively. The measured forward fluorescence intensity of CdSe QDs was enhanced gradually with the thickening of integrated OPC-ex or OPC-em layers. The detected fluorescence intensities via the IOPC-OPC composite exceeded that of the IOPC-Au monolayer by more than twice. The optimal intensity of the double-layer IOPC-Au+OPC-ex composite is higher than that of the IOPC-Au+OPC-em one.

**Fig. 5:**
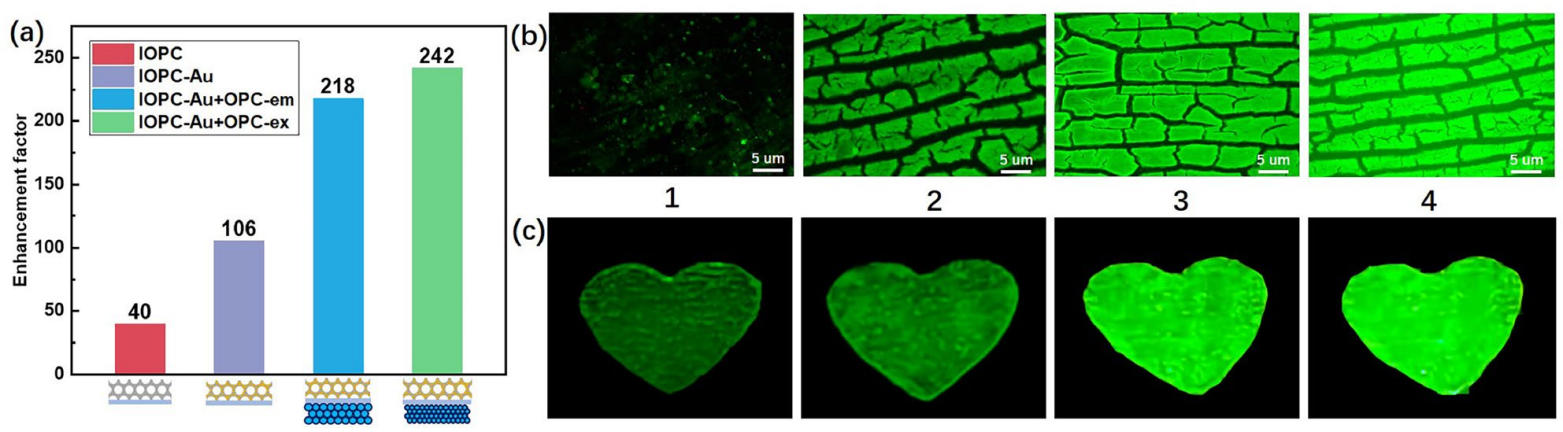
(**a**) Fluorescence enhancement factors of the following structures relative to CdSe QDs drop-casting on quartz substrate: Bare IOPC layer (IOPC), Au-doped IOPC (IOPC-Au), double-layer PC composites with OPC-PBG at emission wavelength 527 nm (IOPC-Au+OPC-em) and OPC-PBG at excitation wavelength 450 nm (IOPC-Au+OPC-em). (**b**) Top-view fluorescence microscope images and (**c**) optical images of a “heart” pattern drawn with CdSe QDs ink on: (1) Quartz substrate, (2) IOPC, (3) IOPC-Au, and (4) IOPC-Au+OPC-ex.

The fluorescence enhancement factor *q* is defined as a ratio of peak emission intensity *I* of CdSe QDs embedded in the composite structure to the reference intensity $I\phantom{0}_0$ of same concentration of CdSe QDs in free space, i.e. $q=I/I_0$. Figure 5a depicts the optimal fluorescence enhancement factors via the above-mentioned PC structures of IOPC, IOPC-Au, IOPC-Au+OPC-em, and IOPC-Au+OPC-ex. The fluorescence intensity was enhanced by: 40-fold (red bar) via the high DOS near the IOPC band edge, 106-fold (purple bar) through the LSPR effect of Au-NPs in the Au-doped IOPC, 218-fold (blue bar) with the OPC-em back-reflecting layer for emission control, 242-fold (green bar) using the OPC-ex back-reflecting layer for excitation confinenent. The high DOS near the band edge enhanced the fluorescent intensity by 40-fold. On this basis, the LSPR effect in the “filling” Au-doped IOPC improved the fluorescent efficiency by 2.65-fold. The integration of OPC reflectors, no matter for excitation or emission reflection, can facilely more than double the fluorescence intensity. Under the synergy effects of near-field LSPR, high-DOS, and PBG-reflection, the double-layer IOPC-Au+OPC-ex composites significantly enhanced the PL efficiency of CdSe QDs by more than 240-fold, far beyond the published counterparts. Under the same exposure of 450 nm excitation light, the PL phenomena of the CdSe QDs infiltrated in different PC configurations were recorded through the microscope and visual observation. The fluorescence micrographs of same dose of CdSe QDs on (1) Quartz plate, (2) IOPC monolayer, (3) Au-doped IOPC monolayer, and (4) IOPC-Au+OPC-ex double-layer are exhibited in Fig. 5b. The top-view optical images of IOPC microstructures (fixed magnification) brighten gradually due to the significantly enhanced fluorescence intensity. In the macroscopic view of Fig. 5c, the “heart” patterns drawn with CdSe QDs ink on different PC structures, corresponding to those in Fig. 5b, proved the fluorescence amplification effects through photonic high-DOS, enhanced-near-field of LSPR, and high-reflection of PBG. The synergy results in a significant boost to the fluorescence radiation efficiency of PL materials, providing important support for future developments of imaging and miniature light sources.

To evaluate the effectiveness of the IOPC-OPC double-layer composites, we compared these performances with the case of a conventional silver (Ag) mirror as a broadband reflector. While the Ag mirror achieved a PL enhancement factor of 210-fold, which is inferior to those of the OPC-em-c2 (218$\times$) and the OPC-ex-c2 (242$\times$) (Supplementary Fig. S5). This discrepancy highlights two critical limitations of metallic reflectors, absorption loss (in visible range 5% -15% ) and limited effect on the light-matter interaction in the upper layer. Mirror-like reflection conserves photon momentum but suppresses scattering and field distribution, resulting in shorter effective light transmission paths in the upper IOPC-Au layer, ultimately limiting the QDs excitation efficiency. In contrast, the OPC-based reflectors offer unique spectral selectivity, by precisely aligning the PBG with either excitation (OPC-ex) or emission (OPC-em) wavelengths. They prolonged the light-QDs interaction process and enhanced the LDOS in the upper IOPC-Au layer, thereby promoting spontaneous radiation and suppressing nonradiative decay of QDs. Furthermore, dielectric OPCs ensure high reflection efficiency with minimal loss. These features collectively enable the IOPC-OPC composites to outperform the broadband metallic reflectors, providing a versatile platform for advanced optoelectronic applications.

In the double-layer IOPC-Au+OPC-ex composite, the breakthrough of 242-fold fluorescence enhancement not only validates the efficacy of the “filling” technique in precisely controlling the spatial arrangement of Au NPs and CdSe QDs but also demonstrates the potential of photonic-plasmonic uniformly hybrid structures for fluorescence manipulation and enhancement. By adjusting the local electromagnetic environment via PC configurations, it is possible to optimize the excitation and emission processes, leading to more efficient and brighter fluorescence. The development of advanced PC composites could revolutionize technologies in precise PL control and high-intensity emission. It would be valuable to explore the synergistic effects of different porous combinations and various fluorescent materials to understand the broader applicability of the “filling” technique. The current work is focused on the specific fluorescence enhancement of CdSe QDs, however, the underlying mechanism can be extended to other fields, such as catalysis, sensing and surface-enhanced Raman spectroscopy, etc. The fabrication techniques of IOPC and OPC components can be optimized for scalability and integration into practical devices. This work provides a promising foundation for the development of advanced optical functional devices with unique properties. By continuing to refine and expand upon this filling technique, future research would unlock new possibilities for PL manipulation and enhancement, with significant implications for a wide range of scientific and technological fields.

## Conclusion

In this work, we have presented an original “filling” technique for substantial fluorescence enhancement of PL materials through the strategic integration of photonic and plasmonic effects within a PC composite system. In this way, the doped Au NPs are well-encapsulated and uniformly dispersed in insulative silica. The prepared porous silica IOPCs have been employed to successfully address the issue of fluorescence quenching between Au NPs and infilled CdSe QDs. The OPC back-layers strengthening excitation or emission reflection further improved light-matter interaction in the porous Au-doped IOPCs, achieving the unprecedented 242-fold fluorescence enhancement. This work advances the PC composite field and sets the stage for wide applications of optical devices in luminescence modulation. The demonstrated strategies are versatile and can be extended to other fluorescent materials, dielectrics, and metal NPs, paving the way for improved performance in various areas, such as photocatalysis, biosensing, imaging, feeble fluorescence signal detection, and display technologies.

## Methods

Materials: Polystyrene microsphere suspension (Casmart Technology), CdSe quantum dots (Xiamen Boer Technology Co., Ltd.), tetraethyl silicate ($\geqslant$99%, purchased from McLean Company), hydrochloric acid (30%, purchased from Beijing Tongguang Fine Chemical Co., Ltd.), anhydrous ethanol ($\geqslant$99%, provided by Tianjin Beilian Company), chloroauric acid ($\geqslant$99%), trisodium citrate ($\geqslant$99%, from Mairida Company). In addition, the water used in this experiment was deionized water.

Preparation of polystyrene OPCs: A series of 10 mL volumetric flasks were prepared to synthesize OPC, then they were filled with 8 mL distilled water and 150 mL polystyrene (PS) suspension. The suspensions were subjected to sonication for 30 min, to ensure uniform dispersion of PS particles. Simultaneously, quartz substrates underwent meticulous cleaning via a plasma cleaner at a power of 150 W for 5 min to eliminate contaminants and enhance their surface quality. Subsequently, the treated quartz substrates were inclined and dipped down into the well-mixed PS suspension and then transferred into a blast drying oven, set at $45 ^{\circ }$C for 12 to 20 hours to settle under the gravity force.

Preparation of silica IOPCs: The polystyrene OPC serves as a sacrificial template. 1 mL tetraethyl orthosilicate (TEOS) was diluted in 10 ml of anhydrous ethanol and stirred for 10 min to ensure thorough mixing. Then, 1 mL of 0.1 mol/L hydrochloric acid (HCl) was dropwise added to the TEOS solution under continuous stirring for 30 min, yielding the uniform silica sol. Then, the silica sol was delicately infiltrated into the void spaces of the pre-prepared opal template under ambient conditions. After the gelation process, the template was moved to a muffle furnace and gradually heated following a ramping schedule of $50 ^{\circ }$C/h until $500 ^{\circ }$C, maintaining for 2 hours to ensure the complete conversion of silica sol into hardened gel. Following this, the sample cools down naturally to room temperature within the furnace.

Integration of Double-layer IOPC-OPC composites: The CdSe QDs solution is infiltrated in the prepared porous Au-doped IOPC on a quartz plane. Then the Au-doped IOPC layer is directly integrated with a prepared OPC on another quartz plane to form the double-layer IOPC-OPC composite, with the IOPC and OPC separated by a quartz plane of 1 mm thickness, as various PC components depicted in Supplementary Fig. S3, such as IOPC, Au-doped IOPC, OPC-ex, and OPC-em can be prepared individually, guaranteeing their respective optical characteristics. In this integration process, these functional components can be freely combined to satisfy different goals.

Characterizations: The fluorescence spectrometer is employed to analyze various PC samples comprehensively, with the results presented in Supplementary Fig. S1 High-resolution scanning electron microscopy (SEM SU8010, Hitachi, Japan) was employed to observe the intricate morphology of Au NPs, CdSe QDs, and PC samples. The transmittance and absorption spectra of the samples were characterized using a UV-visible spectrophotometer (U3900, Hitachi, Japan). The PL spectrum was measured using a steady-state transient fluorescence spectrometer (FLS-1000, Edinburgh, UK), with the optical path for fluorescence spectroscopy measurement shown in Supplementary Fig. S2 The fluorescence intensity of the sample was observed via a fluorescence microscope (OLYMPUS BX53M)

## Supplementary Information

Supplementary Information.

## Data Availability

All data generated or analyzed during this study are included in this published article and its supplementary information file.
