# Supplementary material for: Boosting fluorescence efficiency via filling technique prepared photonic crystal composites
Source: Sci Rep. 2025 Jun 6;15:19990. doi: 10.1038/s41598-025-04296-7 (PMC12144228; doi:10.1038/s41598-025-04296-7)
Supplement: Supplementary file 1 — Supplementary Information. [file 41598_2025_4296_MOESM1_ESM.pdf]

Boosting fluorescence efficiency via filling-technique-prepared photonic crystal composites

Y. T. Qin[1], X. Zhao[1], Y. R. Wang[2], J.X. He[1], Z. Zhu[1], *T. Z. Zhao[1], *G. Y. Dong[1]

1. Center of Materials Science and Optoelectronics Engineering, School of Opto-Electronics, University of Chinese Academy of Sciences, Beijing 100049, China

2. School of Physics, Beijing Institute of Technology, Beijing 100081, China

*E-mail: gydong@ucac.ac.cn (G. Y. Dong); zhaotianzhuo@ucas.ac.cn (T. Z. Zhao)

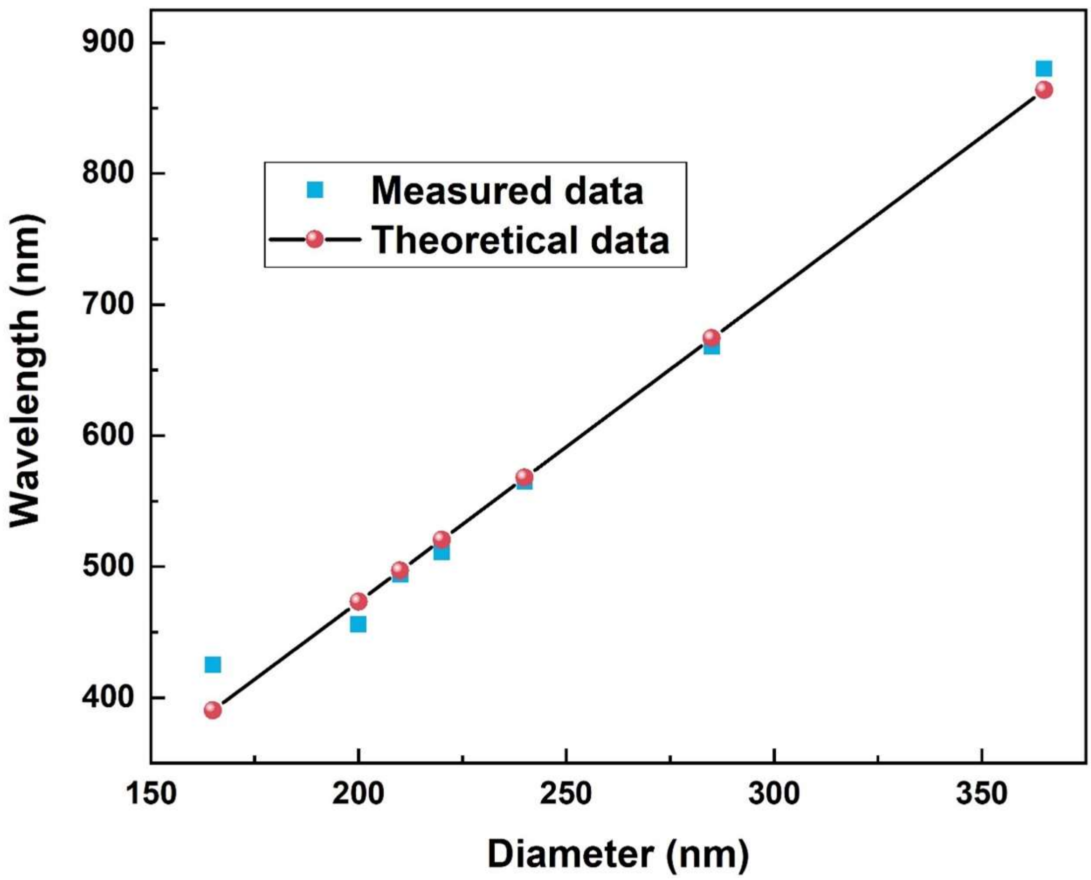

Figure S1. Relationship between diameter of constituent PS microspheres and PBG centeral wavelength of prepared OPC (Corresponding to diameters of 165, 200, 210, 220, 240, 285, and 360 nm)

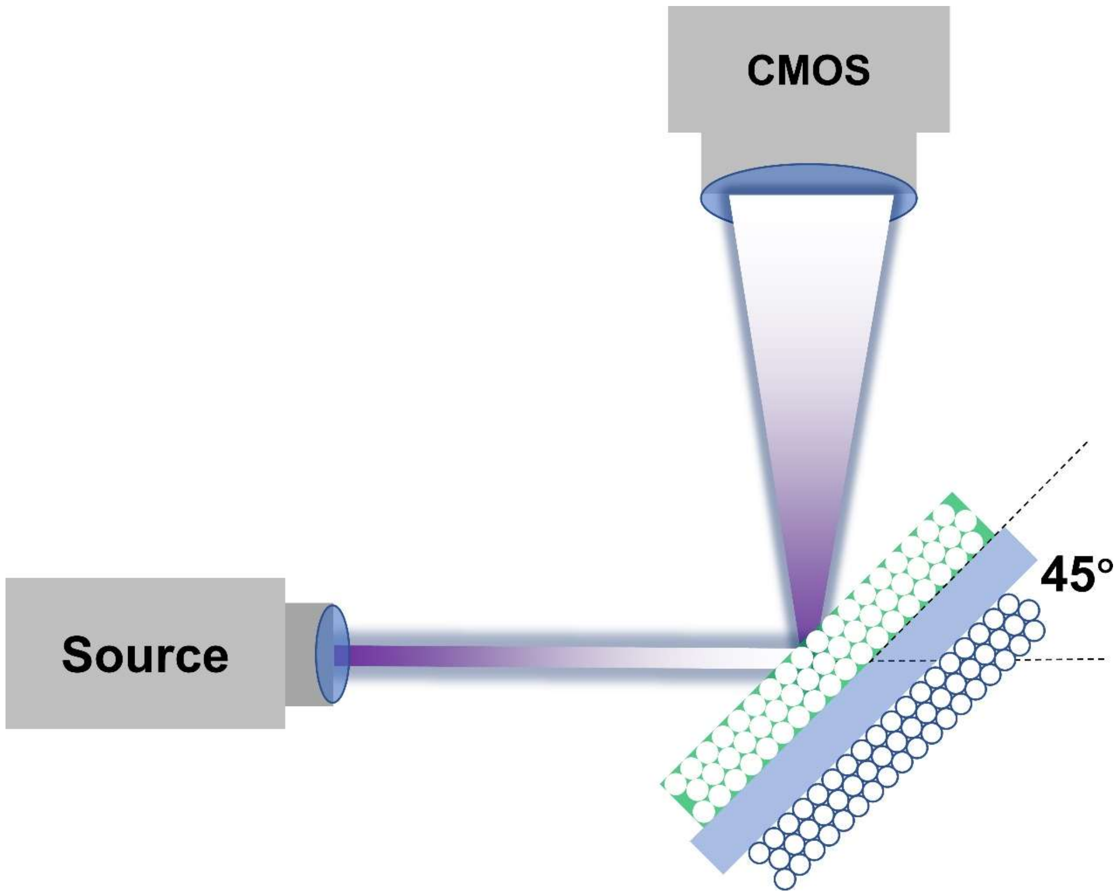

Figure S2. Schematic diagram of the optical path for fluorescence spectroscopy measurement.

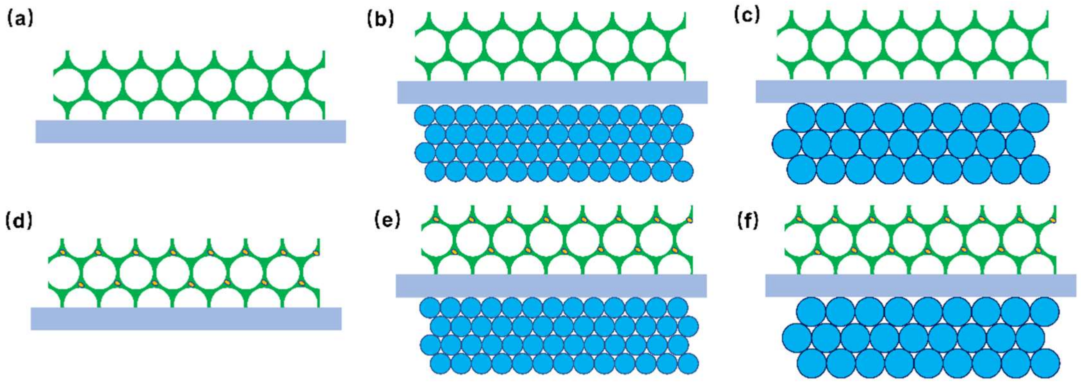

Figure S3. Schematic diagrams of (a) IOPC, (b) IOPC integrated with an OPC excitation reflector (IOPC+OPC-ex), (c) IOPC integrated with an OPC emission reflector (IOPC+OPC-em), (d) Au-doped IOPC, (e) Au-doped IOPC integrated with an OPC excitation reflector (IOPC-Au+OPC-ex), (f) Au-doped IOPC integrated with an OPC emission reflector (IOPC-Au+OPC-em)

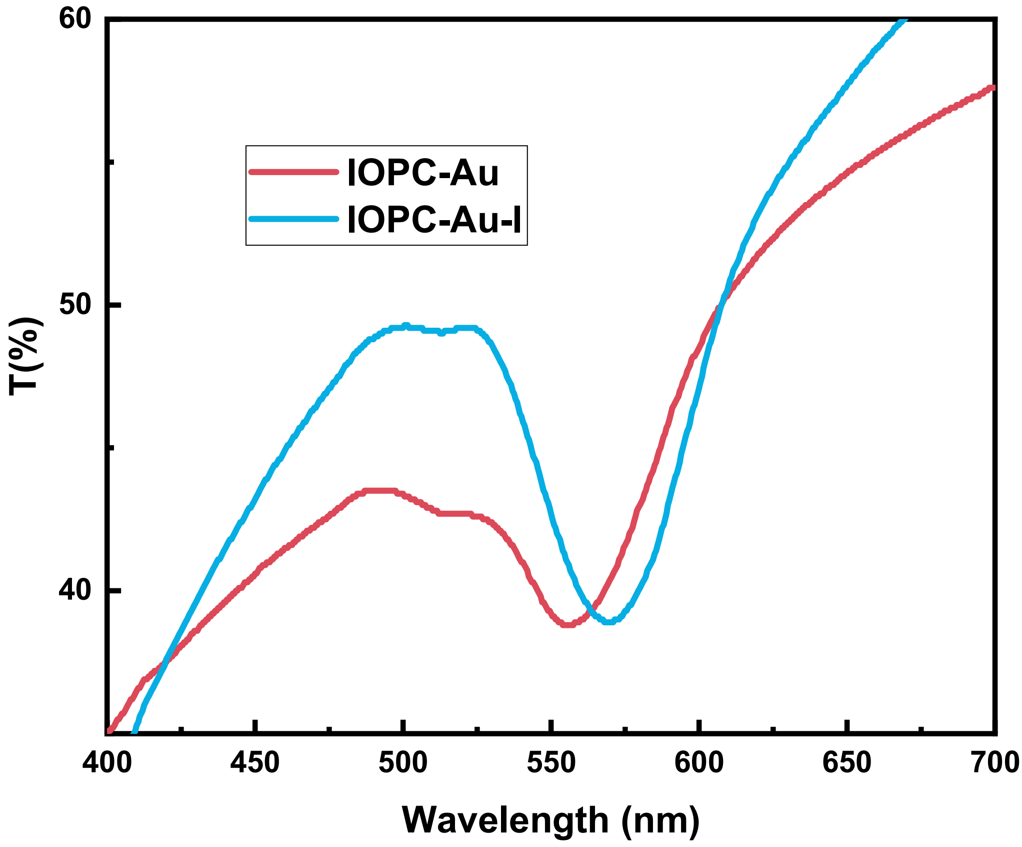

Figure S4. Transmittance spectra of Au-doped IOPC prepared in the one-pot (IOPC-Au-I) and the filling (IOPC-Au) methods, respectively.

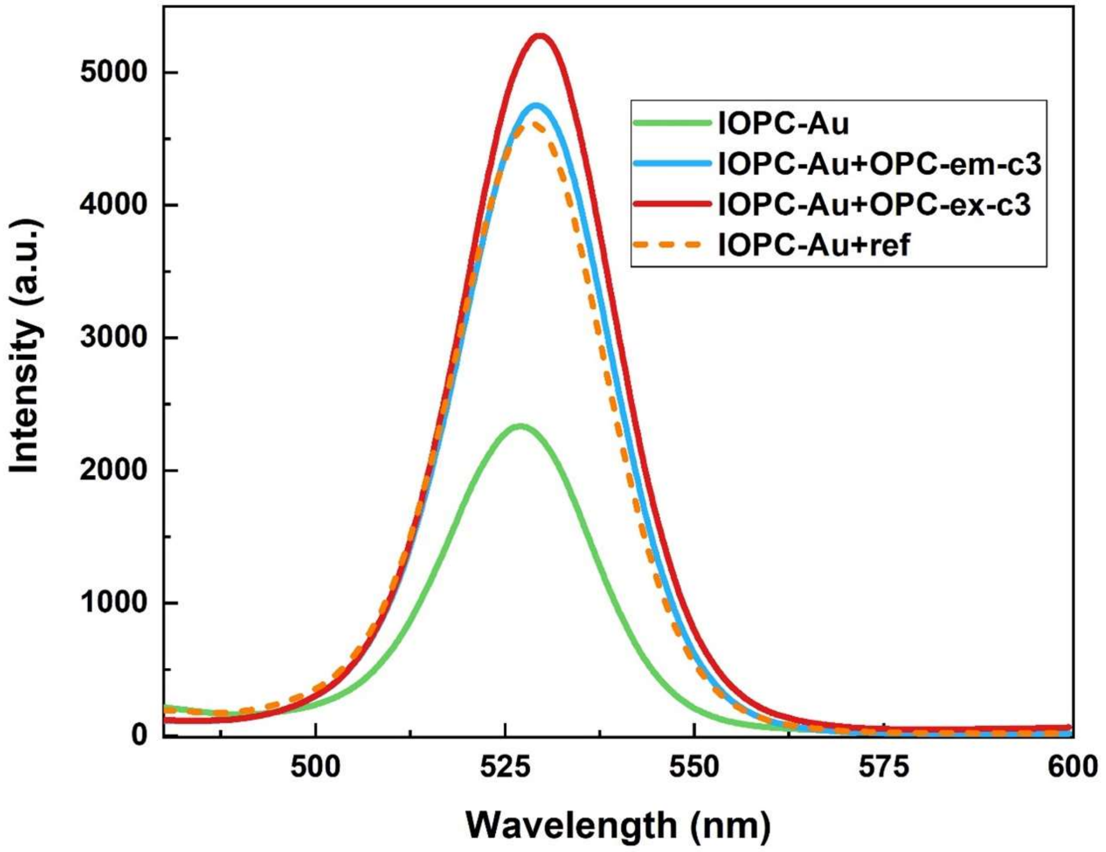

Figure S5. Comparison of fluorescence spectra between the double-layer IOPC-Au+Ag mirror (ref) structure and the two most effective emission-enhancing composite architectures (IOPC-Au+OPC-em-c3 and IOPC-Au+OPC-ex-c3)
